# Kidney transplant artery and vein stenting: 15-year follow-up

**DOI:** 10.1186/s42155-024-00486-y

**Published:** 2024-09-28

**Authors:** Jan H. Peregrin, Daniel Vedlich, Ondřej Viklický

**Affiliations:** 1 Dept. of Diagnostic and Interventional Radiology, Institute for Clinical and Experimental Medicine, Prague, Czech Republic; 2 Dept. Nephrology, Transplant Center, Institute for Clinical and Experimental Medicine, Prague, Czech Republic

**Keywords:** Transplanted kidney, Arterial stenting, Venous stenting

## Abstract

**Background:**

We would like to present an unusual case of simultaneous stenosis of renal graft artery and vein diagnosed four months after transplantation. both treated by stent placement. Our aim is to point at the fact that renal graft venous stenosis is very rarely reported in the literature and – as it is not easy to diagnose by routine US - it could be overlooked. If early detected it can be treated by stent placement.

**Case presentation:**

We present a case of 36-old-male with renal failure who received a kidney graft from deceased donor. The patient experienced delayed graft function. No rejection was found in the biopsy. Four months after transplantation the kidney function deteriorated to sCr 280 µmol/l. Graft artery stenosis together with graft vein stenosis was revealed. Both lesions were dilated with stent placement, the graft function returned to 230 µmol/l and became stable for 10 years. Ten years after stent placement graft function deteriorated to 300 µmol/l. An in stent restenosis of arterial stent was detected. It was successfully dilated by the balloon, the graft function returned to 230 µmol/l and stays stable for another 5 years.

**Conclusions:**

An unusual simultaneous transplanted kidney artery and vein stenosis treated by stent placement is presented. The patient had stable graft function for 15 years after the procedure with one re-intervention on arterial stent.

## Case presentation

We present a case of 36-year-old male suffering from hereditary hypomagnesemia, hypercalciuria and nephrocalcinosis. From July 2007, the patient had undergone hemodialysis therapy complicated by recurrent vascular access stenosis. MTHFR 1298 A˃C heterozygosity was described as the only documented thrombophilic mutation.

In December 2008, the patient received a kidney transplantation from a 46-year-old deceased donor. Cold ischemia time was 24 h. As induction regimen, alemtuzumab therapy was given followed by tacrolimus-based immunosuppression. The patient experienced delayed graft function; initial graft biopsy on POD7 demonstrated acute tubular necrosis, second biopsy on POD21 exhibited interstitial fibrosis and tubular atrophy. No rejection was found in the biopsy. Kidney graft function has remained reduced since then, serum creatinine {sCr} was 232 µmol/l at discharge.

In February 2009, kidney graft function deteriorated (Cr 280 µmol/l) and third graft biopsy revealed borderline changes which were treated by steroid pulses according to our center protocol.

**Fig. 1:**
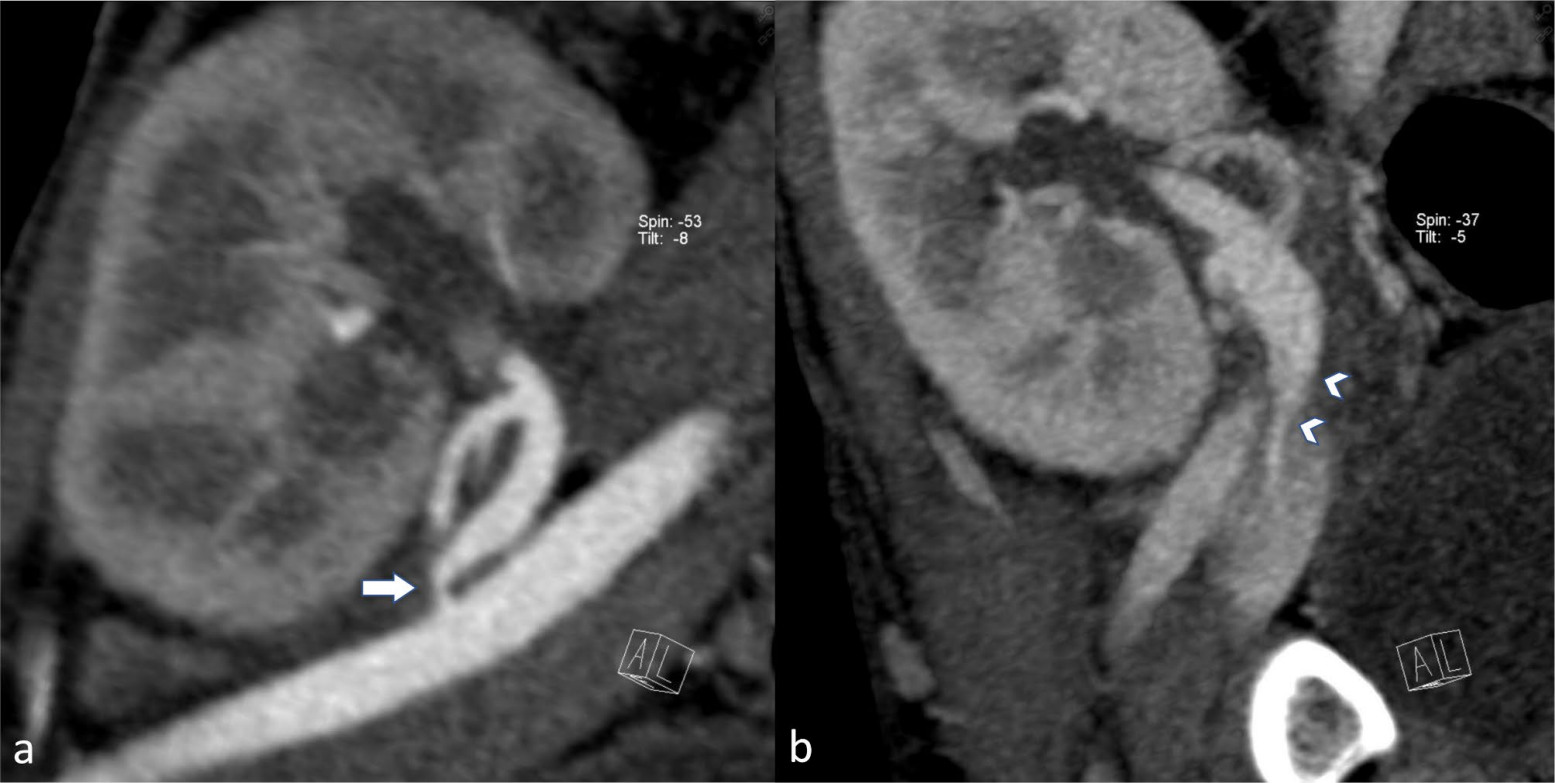
**a:** CTA graft artery stenosis [arrow]. **b:** CTA graft vein stenosis [arrowheads]

**Fig. 2:**
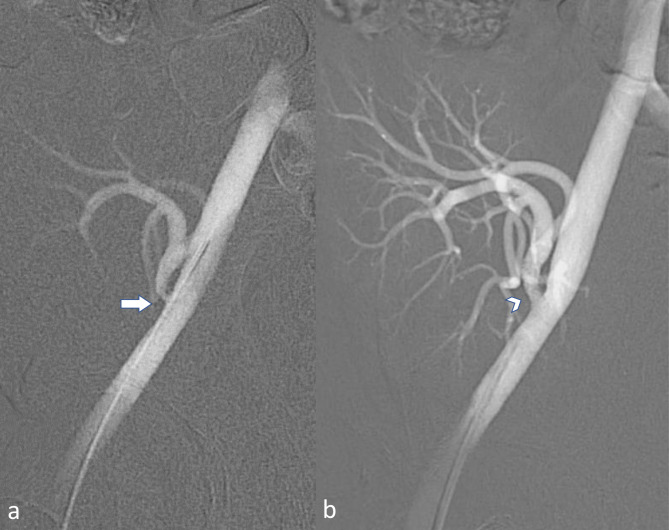
**a:** Graft artery stenosis [arrow] close to end to side anastomosis. **b:** Stent placement to the stenosis location [arrowheads]

Ultrasound examination performed in April 2009 revealed kidney graft artery stenosis confirmed by subsequent CT angiography. The additional analysis of the venous phase of the CT angiogram additionally identified kidney graft vein stenosis [Fig. 1]. Subsequent angiography confirmed 80% graft artery stenosis. Percutaneous transluminal angioplasty {PTA) of the graft artery was performed using a 5 mm/13 mm Pro Kinetic balloon-expandable stent (B Biotronik AG, Bülach, Switzerland) with a favorable post-PTA result (Fig. 2).

**Fig. 3:**
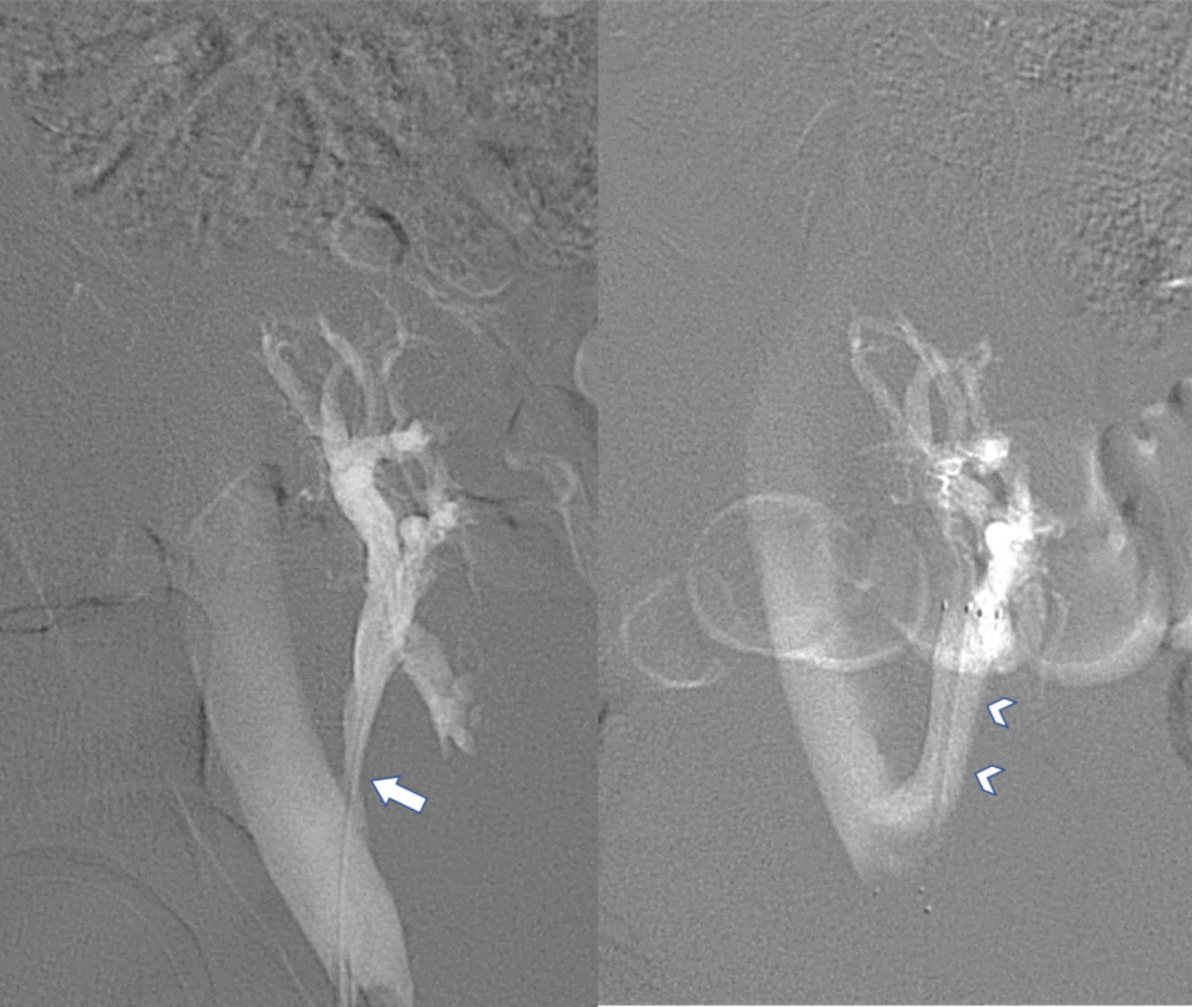
**a:** DSA Graft vein stenosis corresponds with CTA results [arrow]. **b:** Self expandable stent placed to the graft vein [arrowheads]

**Graph 1:**
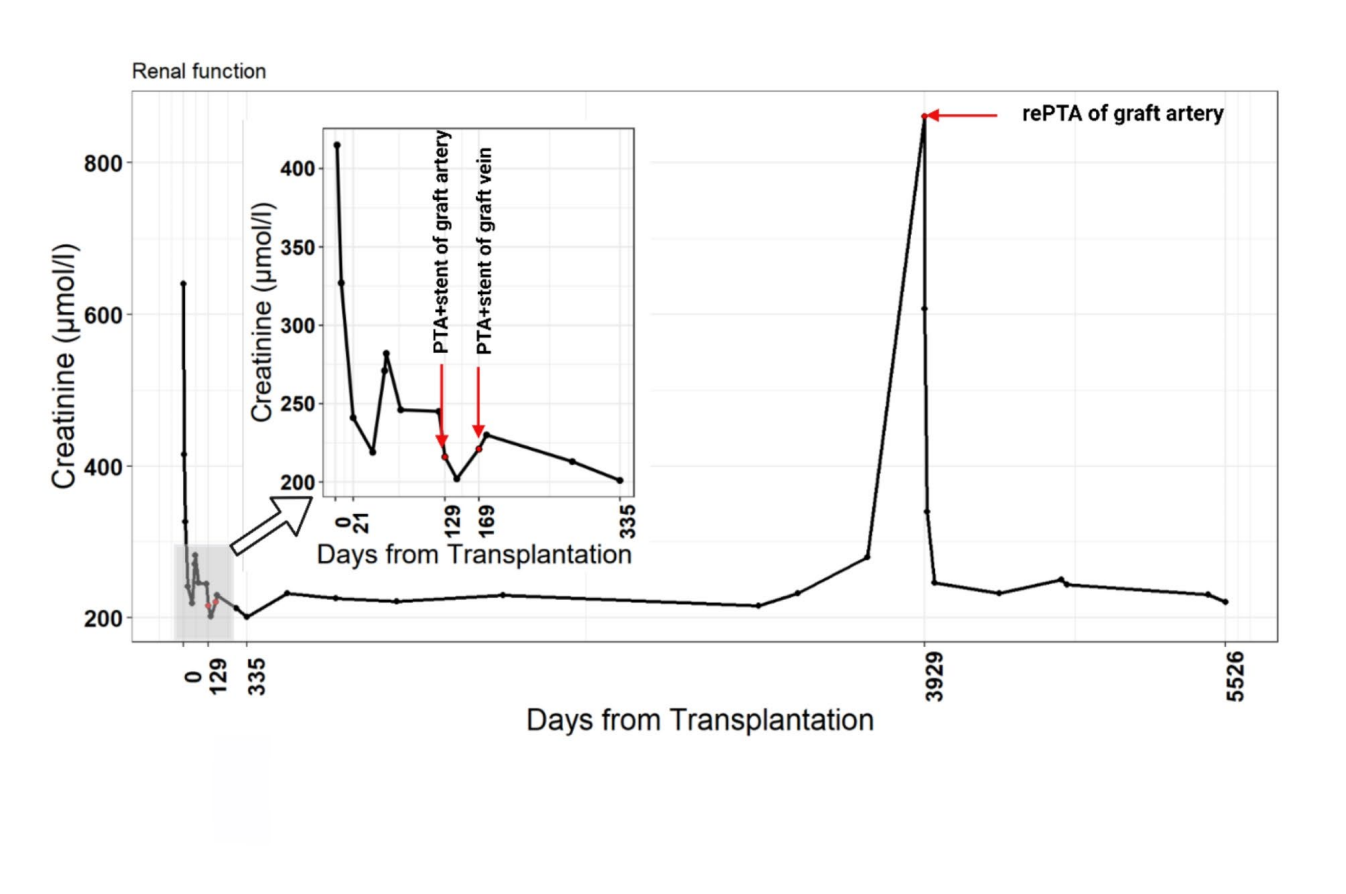
SCr changes during follow-up

In June 2009, phlebography confirmed 70–80% graft vein stenosis which was managed by placement of a 9 mm/40 mm self-expandable Sinus stent {Optimed, Ettlingen, Germany}. The pressure gradients between the graft vein and the external iliac vein were 8 mmHg and 0–1 mmHg prior to and after the procedure, respectively (Fig. 3). In September 2019, the patient experienced further worsening of graft function. Ultrasound assessment revealed arterial in-stent restenosis. Stent dilatation was performed using a 5 mm/30 mm Sterling balloon catheter (Boston Scientific, Natick, MA, USA) with an acceptable result. Kidney graft function remained stable after this procedure (Graph 1).

## Discussion

Transplant renal artery stenosis (TRAS) is among the most common vascular complications of kidney transplantation, with the incidence varying between 1% and 23% [1–4]. While TRAS occurs mostly in the first-year post-transplant, it may also develop much later [5].

Kidney graft function deterioration and severe hypertension have been described to be present in TRAS. However, in many cases the clinical manifestations are nonspecific and subclinical. The diagnosis of TRAS is based established by ultrasound, complemented by CTA and/or MRA. PTA has been considered e the treatment of the first choice and stents have been placed in most cases. While the success rate of TRAS angioplasty is close to 100%, about 10–20% of patients develop restenosis requiring re-angioplasty [3, 5].

Graft vein stenosis, however, is a very rare complication in kidney transplantation. Its diagnosis may be underreported as duplex ultrasound assessment may be inconclusive due to absent or reverse diastolic blood flow. Subsequently graft vein stenosis is complicated by graft vein thrombosis and graft failure. To date, there have been very few reports of kidney graft vein angioplasty. Similar to arterial stenosis, the therapeutic option is angioplasty with stent placement in those cases where technical failure and graft vein kinking are excluded [6, 7]. Published reports described common iliac vein stenosis/thrombosis associated with graft dysfunction and complicated by graft vein thrombosis [8, 9].

In our case, both kidney graft artery and vein stenosis were associated only with marginal deterioration of the graft function. Both stenoses were diagnosed early after transplantation when the expected graft function was not achieved and acute tubular necrosis and borderline rejection were found on biopsies. Both complications can have hemodynamic and functional consequences if they were not treated. Therefore, stable graft function for 10 years post-PTA must be considered as a therapeutic success. The only complication was in-stent restenosis in the kidney graft artery diagnosed at 10 years after initial PTA and stent placement. It was associated with a significant graft dysfunction and was successfully treated by re-PTA [Graph 1].

In conclusion, this case demonstrates that the long-term outcome of kidney graft artery and graft vein stenting may be an acceptable solution especially when the diagnosis is established early after transplantation.

## Data Availability

All clinical data and images are stored in IKEM database.

## References

[1] Aktas S, Boyvat F, Sevmis S, Moray G, Karakayali H, Haberal M. Analysis of vascular complications after renal transplantation. Transpl Proc. 2011;43(2):557–61. 10.1016/j.transproceed.2011.01.007 21440760

[2] Patel NH, Jindal RM, Wilkin T, Rose S, Johnson MS, Shah H, Namyslowski J, Moresco KP, Trerotola SO. Renal arterial stenosis in renal allografts: retrospective study of predisposing factors and outcome after percutaneous transluminal angioplasty. Radiology. 2001;219(3):663–7. 11376251 10.1148/radiology.219.3.r01jn30663

[3] Pini A, Faggioli G, Pini R, Mauro R, Gallitto E, Mascoli C, Grandinetti V, Donati G, Odaldi F, Ravaioli M, La Manna G, Gargiulo M. Assessment and Management of Transplant Renal artery stenosis. A literature review. Ann Vasc Surg. 2022;82:13–29. 35108560 10.1016/j.avsg.2022.01.011

[4] Dimitroulis D, Bokos J, Zavos G, Nikiteas N, Karidis NP, Katsaronis P, Kostakis A. Vascular complications in renal transplantation: a single-center experience in 1367 renal transplantations and review of the literature. Transplant Proc. 2009;41(5):1609-14. 10.1016/j.transproceed.2009.02.077 19545690

[5] Obed A, Zorger DC, Scherer NFS, Krüger MN, Banas B, Krämer B. Severe renal vein stenosis of a kidney transplant with beneficial clinical course after successful percutaneous stenting. Am J Transpl. 2008;8(10):2173–6. 10.1111/j.1600-6143.2008.02356.x 18828776

[6] Mei Q, He X, Lu W, Li Y. Renal vein stenosis after renal transplantation: treatment with stent placement. J Vasc Interv Radiol. 2010;21(5):756–8. 20430299 10.1016/j.jvir.2010.01.037

[7] Cercueil JP, Chevet D, Mousson C, Tatou E, Krause D, Rifle G. Acquired vein stenosis of renal allograft–percutaneous treatment with self-expanding metallic stent. Nephrol Dial Transpl. 1997;12(4):825–6. 10.1093/ndt/12.4.825 9141026

[8] Jones G, Tibballs J, Al-Akraa M, Sweny P. Iliac vein stenosis as a reversible cause of renal transplant dysfunction. Nephrol Dial Transpl. 2004;19(9):2415–6. 10.1093/ndt/gfh225 15299107

[9] Fava M, Loyola S, Flores P, Del Campo F. External iliac vein thrombosis after renal transplantation: treatment by thrombolysis and stent placement: a case report. Transplantation. 1997;64(6):928–30. 9326424 10.1097/00007890-199709270-00025

